# Endothelial Sprout Formation Is Regulated by Substrate Stiffness and Notch Signaling

**DOI:** 10.3390/ijms26073155

**Published:** 2025-03-28

**Authors:** Maibritt Kretschmer, Angelika M. Vollmar, Stefan Zahler

**Affiliations:** Department of Pharmacy, Ludwig-Maximilians-University, Butenandtstr. 5, 81377 Munich, Germany; maibritt.kretschmer@medicalschool-hamburg.de (M.K.);

**Keywords:** Notch, stiffness, angiogenesis, Dll4

## Abstract

Angiogenesis, the process of vessel formation from pre-existing ones, is modulated by the local stiffness of the extracelluar matrix. We have previously shown that Notch signaling, a key pathway in angiogenesis, responds to substrate stiffness in endothelial cells. In the current work, we investigate the contribution of Notch signaling in angiogenesis-related in vitro assays by using VEGF and Notch inhibitors as perturbations. In addition, we investigate Notch signaling in relation to the stiffness of the respective endothelial microenvironment. While the tube formation assay on Matrigel is clearly influenced by substrate stiffness, Notch signaling seems to play no major role in this context. In contrast, spheroid sprouting is influenced by stiffness as well as Notch signaling; with decreasing stiffness, both sprouting and Notch signaling are increased. This finding adds a functional aspect to the mechanosensitivity of Notch signaling.

## 1. Introduction

Angiogenesis, the process of vessel formation from pre-existing ones, is physiologically and pathophysiologically of great importance, e.g., during wound healing, tissue growth, or tumor growth [1]. Over the last years, there has been an increase in the awareness that, in addition to biochemical gradients, mechanical cues from the microenvironment play a key role for the development and the morphology of blood vessels [2,3,4]. Initially, the focus was on mechanical forces exerted by blood flow [5,6]. However, it soon turned out that the stiffness and architecture of the extracellular matrix are of critical importance [4,7,8].

There are some canonical mechanosensitive signaling pathways, which are involved in the transduction of mechanical cues to changes in cellular behavior, like the SRF/MRTF [9] axis or Hippo/YAP signaling [10]. These signaling pathways rely on a translocation of modulators of transcription from the cytoplasm to the nucleus upon the remodeling of the cytoskeleton. MRTF [11,12], as well as YAP [13], has already been shown to play an important role during angiogenesis. Interestingly, for Notch signaling, a pathway of central importance for angiogenesis; there is only limited information on its modulation by mechanics during this process.

In the context of angiogenesis, Notch signaling mainly regulates the selection of tip and stalk cells via the interaction of Notch1 and its ligand Dll4 [14], with an inhibition of this pathway leading to unproductive sprouting behavior [15]. It has recently been shown that Notch signaling is modulated by mechanical cues [16]. The cellular and molecular source for this mechanosensitivity seem to be the formation of catch bonds between Notch receptors and their ligands, which strengthen under tension, and the influence on the internalization of the cleaved receptors [17,18]. We have previously shown that matrix stiffness influences the endothelial tube formation of a soft Matrigel^®^ (Corning, NY, USA) matrix [4], and that it also regulates Notch activity in endothelial cells [19] in a 2D setting. Here, we investigate whether substrate stiffness also influences endothelial sprouting behavior and Notch activity in a more complex 3D environment.

## 2. Results and Discussion

**Figure 1:**
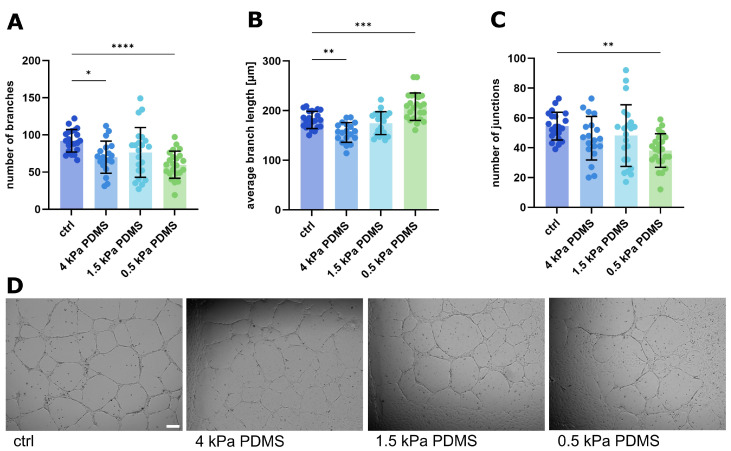
Changes in tube structures after the stiffness modification of the underlying matrix. (**A**–**C**) Analysis of tube structures after the hydrogel printing assay with HUVEC cells. A thin Matrigel layer enabling tube formation was added onto PDMS substrates with the different stiffnesses indicated above. Tube formation was then influenced by the mechanical changes. Data from three independent experiments, each with at least triplicates, are shown in scatter plots as mean ± s.d. * *p* < 0.1; ** *p* < 0.01; *** *p* < 0.001; **** *p* < 0.0001 (one-way ANOVA followed by Tukey’s multiple comparison test). (**A**) Number of branches. (**B**) Average branch length in µm. (**C**) Number of meshes. (**D**) Representative images of tube structures after performing the tube formation assay; incubation for 24 h. Scale bar: 100 µm.

**Figure 2:**
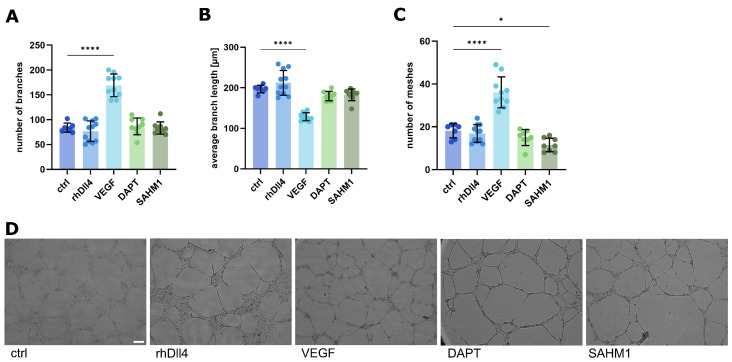
Changes in tube structures after chemical influence of the Notch signaling pathway. (**A**–**C**) Analysis of tube structures after the tube formation assay with HUVEC cells. Tube formation was influenced by the addition of the compounds listed above in the medium during cell seeding. Data from three independent experiments, each with at least triplicates, are shown in scatter plots as mean ± s.d. * *p* < 0.1; **** *p* < 0.0001 (one-way ANOVA followed by Tukey’s multiple comparison test). (**A**) Number of branches. (**B**) Average branch length in µm. (**C**) Number of meshes. (**D**) Representative images of tube structures after performing a tube formation assay; incubation for 24 h. Scale bar: 100 µm.

We previously described the influence of matrix stiffness on endothelial tube formation in the classical Matrigel^®^ tube formation assay [4]. Therefore, we first investigated the influence of a range of substrate stiffnesses between 4 kPa and 0.5 kPa on tube formation (Figure 1). With decreasing stiffness, the network morphology changes, i.e., the number of junctions and branches in the network decreases (Figure 1A,C), while the average branch length increases (Figure 1B). This underscores the mechanical influence on the process of tube formation, which we already described [4]. In order to dissect the potential contribution of Notch signaling to this phenomenon, we then used a stimulation with the Notch ligand Dll4 or VEGF, which has been shown to modulate Notch signaling [20], as well as the inhibition of Notch by the well-established pharmacological inhibitors DAPT and SAHM1. Interestingly, though the stimulation with VEGF increased the number of branches and decreased the average branch length (Figure 2A–D), the stimulation of the Notch signaling pathway with recombinant human Dll4 or inhibition of Notch signaling with inhibitors did not consistently change the network morphology (Figure 2A–D). This might indicate that, in this setting, the morphogenic process of tube formation is predominantly regulated by local physical cues and not so much by biochemical signaling events, like our previous findings suggested [4]. Accordingly, this model seems not to be a good choice for studying the influence of Notch signaling in soft matrices. Though the assay has been used as a standard in vitro system to study angiogenesis for many years [21], its limitations have been increasingly recognized [22].

As an alternative model, we next investigated vascular sprouting from endothelial spheroids in collagen gels, and the influence of VEGF, or rhDll4 and Notch signaling inhibitors.

**Figure 3:**
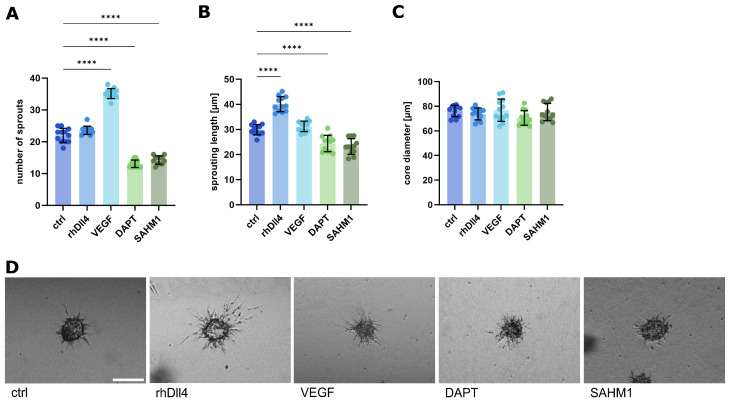
Changes in spheroid sprouting behavior after the chemical influence of the Notch signaling pathway. (**A**–**C**) Analysis of sprouting behavior after spheroid sprouting assay with HUVEC cells. Spheroid sprouting was influenced by the addition of the compounds listed above in the medium during spheroid embedding. Data from three independent experiments, each with at least triplicates, are shown in scatter plots as mean ± s.d. **** *p* < 0.0001 (one-way ANOVA followed by Tukey’s multiple comparison test). (**A**) Number of sprouts. (**B**) Average sprouting length in µm. (**C**) Core diameter of the spheroid bodies. (**D**) Representative images of spheroids after performing a spheroid sprouting assay; incubation for 24 h. Scale bar: 100 µm.

Stimulation with VEGF increased the number of sprouts (Figure 3A), but did not change the length of the single sprouts (Figure 3B). Activation of Notch signaling with rhDll4 increased the length of sprots (Figure 3B), but did not change the degree of sprouting (Figure 3A). Inhibition of Notch signaling by the inhibitors DATP or SAHM1 reduced both the sprout number and the length (Figure 3A,B). Importantly, the core diameter of the endothelial spheroids was not changed by any of the treatments (Figure 3C), indicating that the treatments did not influence proliferation or cell death. This provides clear evidence that Notch signaling plays a role in sprouting behavior in a 3D fibrous hydrogel model.

**Figure 4:**
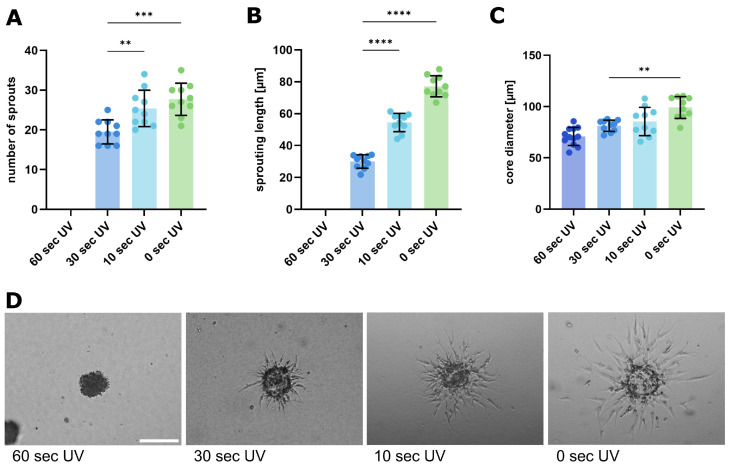
Changes in spheroid sprouting behavior after stiffness modification of the surrounding matrix. (**A**–**C**) Analysis of sprouting behavior after spheroid sprouting assay with HUVEC cells. A photoinducible collagen I hydrogel was applied as a surrounding matrix. Spheroid sprouting was influenced by stiffness changes due to different exposure times to UV light as indicated above. Data from three independent experiments, each with at least triplicates, are shown in scatter plots as mean ± s.d. ** *p* < 0.01; *** *p* < 0.001; **** *p* < 0.0001 (one-way ANOVA followed by Tukey’s multiple comparison test). (**A**) Number of sprouts. (**B**) Average sprouting length in µm. (**C**) Core diameter of the spheroid bodies. (**D**) Representative images of spheroids after performing a spheroid sprouting assay; incubation for 24 h. Scale bar: 100 µm.

Next, we studied the influence of matrix stiffness on this sprouting process, and the contribution of Notch signaling. Collagen gels of varying stiffness were created by using a photo-curable collagen [23] and illumination with UV light for different times. Measurements of gel stiffness after 60 s, 30 s, 10 s, and no illumination, as well as the gel fibrillar structure, are shown in Supplementary Figure S1. The resulting stiffness values range from 0.05 to 0.8 kPa. The highest matrix stiffness (0.8 kPa) did not enable sprouting, while the number of sprouts and sprout length increased inversely with the decreasing matrix stiffness (Figure 4A,B). Even the core diameters increased with decreasing stiffness, indicating reduced mechanical confinement (Figure 4C). This shows a direct correlation between matrix stiffness and endothelial sprouting intensity, which is in good accordance with the previous studies [24].

**Figure 5:**
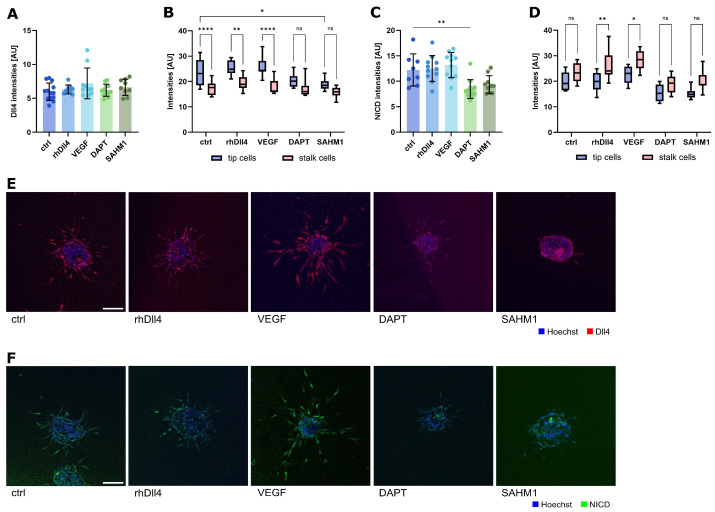
Expression of Dll4 and NICD in sprouted spheroids after the chemical influence of the Notch signaling pathway. (**A**–**D**) Notch ligand (Dll4) and receptor (NICD) intensities (a.u. arbitrary units) in sprouted HUVEC spheroids. Spheroid sprouting was influenced by the addition of the compounds listed above in the medium during spheroid embedding. Intensities were analyzed in the complete spheroid (overall) as well as single cells (tip and stalk cells). (**A**) Overall Dll4 intensity. Data from three independent experiments, each with at least triplicates, are shown in a scatter plot as mean ± s.d. (**B**) Dll4 intensities in tip and stalk cells shown in a violin plot as min to max. * *p* < 0.1; ** *p* < 0.01; **** *p* < 0.0001; ns, not significant (one-way ANOVA followed by Šídák’s multiple comparisons test). (**C**) Overall NICD intensity. Data from three independent experiments, each with at least triplicates, are shown in a scatter plot as mean ± s.d. ** *p* < 0.01 (one-way ANOVA followed by Tukey’s multiple comparison test). (**D**) NICD intensities in tip and stalk cells shown in a violin plot as min to max. * *p* < 0.1; ** *p* < 0.01; ns, not significant (one-way ANOVA followed by Šídák’s multiple comparisons test). (**E**,**F**) Representative images of sprouted spheroids; incubation for 24 h. Cells are stained for nuclei (blue) and Dll4 (red) or NICD (green). Scale bar: 100 µm.

To validate the staining of components of Notch signaling (Dll4 and cleaved Notch receptor) as surrogate parameters for Notch activity, we investigated the intensities of Dll4 and NICD staining overall, and separated this into tip and stalk cells, respectively, after the incubation of endothelial spheroids with rhDLL4, VEGF, or the Notch inhibitors DAPT or SAHM1 (Figure 5). While the overall staining intensity was not significantly changed by the different treatments (Figure 5A,C), a clear difference between the tip and stalk cells is visible for Dll4 and NICD under control conditions (Figure 5B,D). As was to be expected, Dll4 levels were higher in the tip cells and NICD was higher in the stalk cells. Activation of Notch signaling by rhDll4 or VEGF did not change the distribution of Dll4, but increased NICD in stalk cells, as compared to the tip cells (Figure 5B,D). Inhibition of Notch signaling by DATP or SAHM1 obviously levelled out the differences between the tip and stalk cells for both parameters. Accordingly, the measurement of Dll4, and even more so NICD differentially in the tip and stalk cells, seems to provide an insight into Notch signaling in this model.

**Figure 6:**
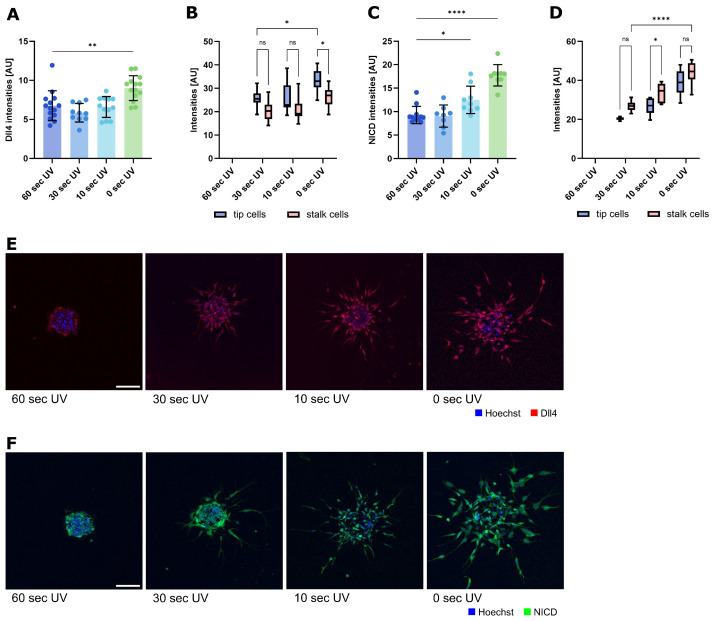
Expression of Dll4 and NICD in sprouted spheroids after stiffness modification of the surrounding matrix. (**A**–**D**) Notch ligand (Dll4) and receptor (NICD) intensities (a.u. arbitrary units) in sprouted HUVEC spheroids. A photoinducible collagen I hydrogel was applied as a surrounding matrix. Spheroid sprouting was influenced by stiffness changes due to different exposure times to UV light as indicated above. Data from three independent experiments, each with at least triplicates, are shown in a scatter plot as mean ± s.d. ** *p* < 0.01 (one-way ANOVA followed by Tukey’s multiple comparison test). (**B**) Dll4 intensities in tip and stalk cells shown in a violin plot as min to max. * *p* < 0.1; ns, not significant (one-way ANOVA followed by Šídák’s multiple comparisons test). (**C**) Overall NICD intensity. Data from three independent experiments, each with at least triplicates, are shown in a scatter plot as mean ± s.d. * *p* < 0.1; **** *p* < 0.0001 (one-way ANOVA followed by Tukey’s multiple comparison test). (**D**) NICD intensities in tip and stalk cells shown in a violin plot as min to max. * *p* < 0.1; **** *p* < 0.0001; ns, not significant (one-way ANOVA followed by Šídák’s multiple comparisons test). (**E**,**F**) Representative images of sprouted spheroids; incubation for 24 h. Cells are stained for nuclei (blue) and Dll4 (red) or NICD (green). Scale bar: 100 µm.

When we performed the same analysis of Dll4 and NICD levels in spheroids embedded in collagen gels of varying stiffness (Figure 6), we observed overall increases in both Dll4 and NICD with decreasing matrix stiffness (Figure 6A,C), indicating increased Notch activity with decreasing stiffness. At higher substrate stiffness differentiation between the tip and stalk cells seems to be less pronounced concerning Dll4 (Figure 6B). Concerning NICD, the difference between tip and stalk cells seems to be the highest with intermediate stiffness (Figure 6D). Thus, Notch signaling seems to be influenced by substrate stiffness during endothelial sprouting. UV irradiation during the polymerization process itself might influence cell behavior. Thus, we tested cell survival after UV treatment and found no significant changes up to 30 s of UV light use. At the longest incubation time (60 s), there is a certain loss of cell numbers 24 h after treatment (Supplementary Figure S2). The results at this time point have, therefore, to be interpreted with caution.

As mentioned above, other signaling pathways, like MRTF/SRF or YAP, have previously been shown to be mechanosensitive and to play an important role in angiogenesis [11,12,13]. Since, for the YAP pathway, a crosstalk to Notch signaling has been reported [25,26], it is experimentally extremely difficult to distinguish between the direct and indirect effects of substrate stiffness on Notch signaling. Though our results should be, consequently, interpreted with care, they still indicate that the endothelial sprouting model in a collagen gel is a good assay system for further in-depth analysis, e.g., based on single-cell transcriptomics or proteomics.

## 3. Materials and Methods

### 3.1. Cell Culture

Human umbilical vein endothelial cells (HUVECs) were purchased from PromoCell (Heidelberg, Germany) and cultured in endothelial cell growth medium (ECGM) obtained from PELO Biotech (Planegg, Germany), supplemented with the ECGM kit, 10% heat-inactivated fetal calf serum (FCS, PAA Laboratories GmbH, Pasching, Austria), 1% penicillin/streptomycin (10,000 U/mL, PAN Biotech, Aidenbach, Germany), and 1% amphotericin B (250 µg/mL). Cells were maintained at 37 °C in a high-humidity environment with 5% CO_2_ and were used in experiments at passage 6.

For optimal adhesion during cultivation, all applied surfaces were pre-coated with collagen G (10 µg/mL in PBS, MATRIX BioScience GmbH, Mörlenbach, Germany).

### 3.2. Antibodies, Compounds, and Staining Reagents

The following primary antibodies used in this study were directed against: Cleaved Notch1 (Val1744) rabbit mAB IgG, 4147 (Cell Signaling Technology, Danvers, MA, USA); DLL4 mouse mAB IgG, ab61031 (abcam, Cambridge, UK). The following secondary antibodies were applied in this study: Alexa Fluor 488-conjugated goat anti-mouse IgG (H+L), A-11001; Alexa Fluor 647-conjugated chicken anti-rabbit IgG (H+L), A-21443 (all from Thermo Fisher Scientific, Waltham, MA, USA).

Recombinant Human DLL4 was obtained from R&D Systems (Wiesbaden, Germany) and used at a working concentration of 1 µg/mL. Recombinant human vascular endothelial growth factor (VEGF) 165 was purchased from PeproTech (Rocky Hill, NJ, USA) and applied at 25 ng/mL. DAPT was purchased from Sigma Aldrich and used at a working concentration of 25 µM. SAHM1 was obtained from R&D Systems (Wiesbaden, Germany) and applied at 10 µM. Hoechst 33342 was purchased from Sigma Aldrich, solved in PBS, and applied at a final concentration of 10 µg/mL. FluorSave™ Reagent was purchased from Merck Millipore (Darmstadt, Germany).

### 3.3. PDMS Preparation

PDMS base elastomer and curing agent (Dow Corning, Midland, MI, USA) were mixed in specific ratios to achieve desired stiffnesses: 10:1 for 70 kPa, 50:1 for 1.5 kPa, and 75:1 for 0.5 kPa. The mixture was degassed in a desiccator for 15–20 min to remove air bubbles, and defined volumes were added to cell culture dishes. PDMS substrates were polymerized at 60 °C for 20 h, then hydrophilized by plasma cleaning at 0.3 mbar O_2_ for 3 min before use. Stiffness measurements for the different mixing ratios are depicted in Supplementary Figure S1A.

### 3.4. Hydrogel Preparation

The hydrogel used was PhotoCol (Advanced BioMatrix, Carlsbad, CA, USA) at a concentration of 4 mg/mL. All preparations were carried out on ice. Lyophilized methacrylated collagen was initially reconstituted in 20 mM acetic acid. Following the manufacturer’s instructions, the collagen solution was first combined with a neutralizing solution, followed by the addition of a photoinitiator based on the volume of collagen used. The prepared collagen gel was then utilized to embed spheroids for the spheroid sprouting assay.

### 3.5. Tube Formation Assay

Tube formation assays were conducted in µ-slides angiogenesis (ibiTreat) from ibidi (Gräfelfing, Germany). Each inner well was filled with 10 µL of Matrigel (growth factor reduced, phenol red free; Corning, New York, NY, USA). Following polymerization, 50 µL of cell suspension, prepared at a concentration of 200,000 cells/mL, was added to the upper well and incubated for 24 h. For experiments involving treatment, compounds were diluted to the specified concentration in the cell suspension before seeding onto the Matrigel. Tube formation was observed and recorded using a Leica DMi1 microscope (Leica Microsystems, Wetzlar, Germany) equipped with a 4× phase contrast objective.

### 3.6. Hydrogel Printing with the Subsequent Tube Formation Assay

Hydrogel printing was carried out in 8-well slides (untreated; ibidi, Gräfelfing, Germany). PDMS substrates with different stiffnesses were produced and polymerized in these slides. The PDMS was activated in the plasma cleaner. A total of 1 μL of Matrigel (growth factor reduced, phenol red free; Corning, New York, NY, USA) was added to the center of the well and evenly distributed using a 5 mm × 5 mm PDMS stamp (70 kPa, not activated). The Matrigel was polymerized for 30 min at 37 °C, 5% CO_2_. To release the stamp, the well was flooded with PBS and the stamp was removed with tweezers. Following this, cell seeding and incubation were carried out in the same way as for regular tube formation.

### 3.7. Spheroid Sprouting

Spheroids were generated using the hanging drop method by pipetting 1000-cell drops with a 20% methyl cellulose solution onto the lids of 10 mm petri dishes and incubating for 24 h. Collected spheroids were centrifuged, then resuspended in FCS. A mixture of a collagen and spheroid solution at a 2:1 ratio was pipetted as a dome into hydrophilized 8-well slides (untreated; ibidi, Gräfelfing, Germany). After polymerization by UV light (254 nm) for the respective time periods, ECGM with 25 nM VEGF was added, and samples were incubated for 24–48 h. For experiments involving treatment, compounds were added to the collagen–spheroid mixture before embedding. Spheroid sprouting was visualized using a Leica DMi1 microscope with a 4× phase contrast objective or analyzed by immunofluorescence staining.

### 3.8. Immunofluorescence Staining and Laser Scanning Confocal Microscopy

For immunostaining spheroids embedded in collagen, hydrogels were washed with phosphate buffered saline containing Ca_2+_ and Mg_2+_ (PBS+) for 10 min, fixed with 4% methanol-free formaldehyde (Thermo Fisher Scientific, Waltham, MA, USA) in PBS for 30 min, then washed again with PBS for 10 min. Permeabilization was performed using 0.1% Triton X-100 in PBS for 30 min, followed by another PBS wash. Blocking was performed with 5% BSA (Roth, Karlsruhe, Germany) in PBS for 3 h at 4 °C. Spheroids were incubated overnight at 4 °C with the primary antibody in PBS with 1% BSA (1:200), then washed six times for 10 min each with PBS containing 1% BSA. Secondary antibody (1:400) and Hoechst 33342 (10 µg/mL) for nuclear staining were added overnight at 4 °C in PBS with 1% BSA. Final washes included two 20 min washes with PBS containing 1% BSA, one 20 min PBS wash, and sealing with a FluorSave mounting medium.

Confocal images were captured using a Leica TCS SP8 microscope (Leica Microsystems, Wetzlar, Germany) with an HCX PL APO 10x/0.40 CS dry objective, equipped with photomultiplier (PMT) or HyD detectors, and operated via LAS X core software (version 5.3.0). Images were acquired in a sequential scanning mode with two frames per channel at a scanning speed of 400 Hz and a pinhole size of 1.0 airy units. Excitation was achieved using laser lines at 405 nm, 488 nm, and 647 nm.

### 3.9. Data Analysis and Statistics

All confocal images were analyzed using ImageJ version 1.54f. Intensity ratios nuclear/cytoplasmatic were evaluated with the Intensity Ratio Nuclei Cytoplasm Tool plugin for ImageJ. Expression patterns were determined by the segmentation and skeletonizing of the images.

Data were obtained from three independent experiments and are presented as mean ± SEM. Statistical analyses were conducted using GraphPad Prism 10.2.3, employing ordinary one-way ANOVA with Dunnett’s multiple comparison test and two-way ANOVA with Tukey’s multiple comparison test.

## Data Availability

The raw data supporting the conclusions of this article will be made available by the authors on request.

## Supplementary Materials

The following supporting information can be downloaded at: https://www.mdpi.com/article/10.3390/ijms26073155/s1.
