# Supplementary figures and images for: Endothelial Sprout Formation Is Regulated by Substrate Stiffness and Notch Signaling
Source: Int J Mol Sci. 2025 Mar 28;26(7):3155. doi: 10.3390/ijms26073155 (PMC11988845; doi:10.3390/ijms26073155)

**A**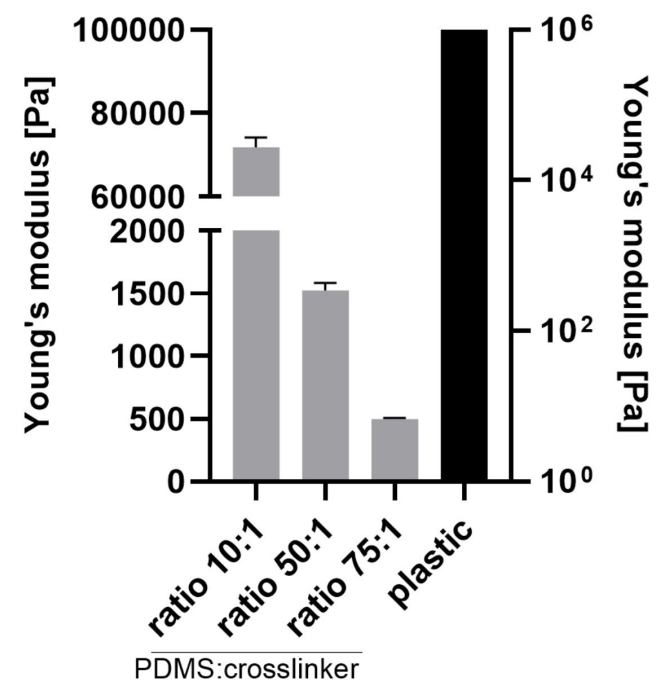**B**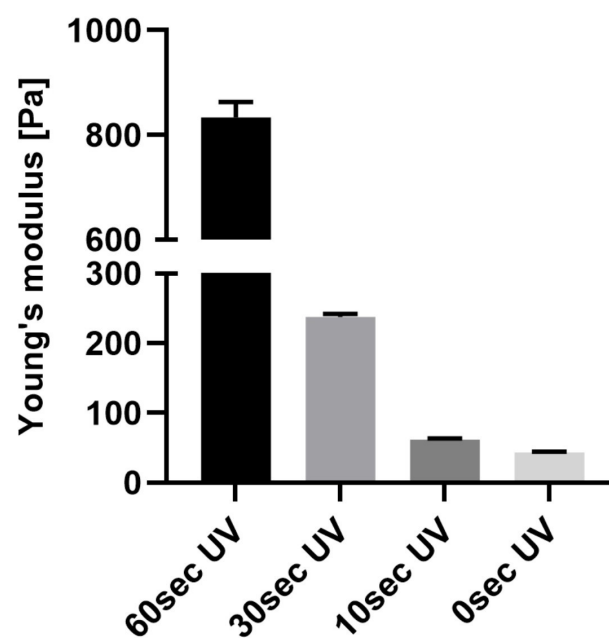**C**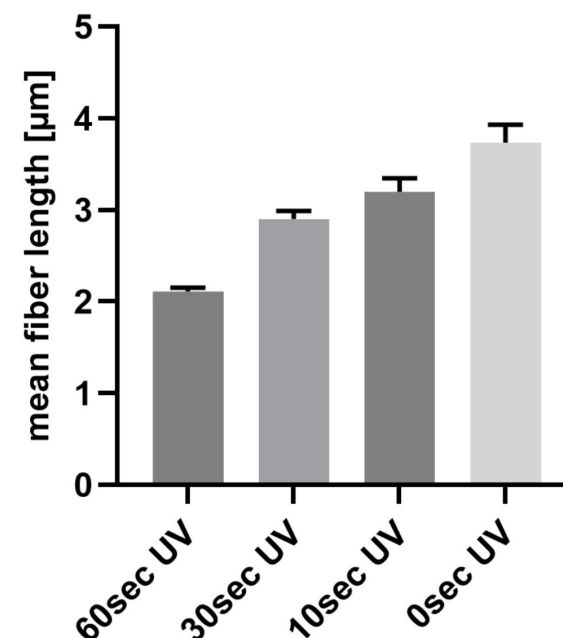**D**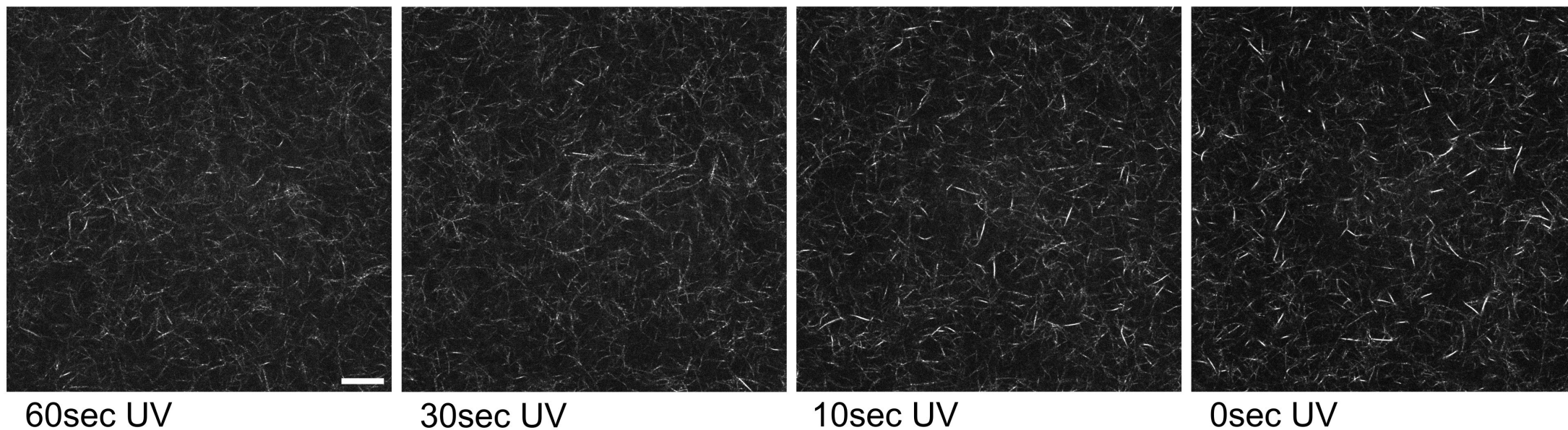

Supplement: Supplementary file 1 [file ijms-26-03155-s001.zip › Supplementary Figure S1.pdf]

**A**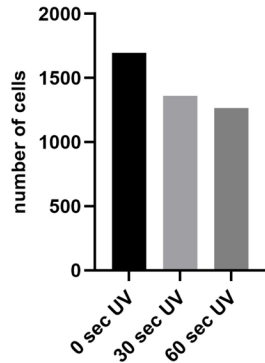**B**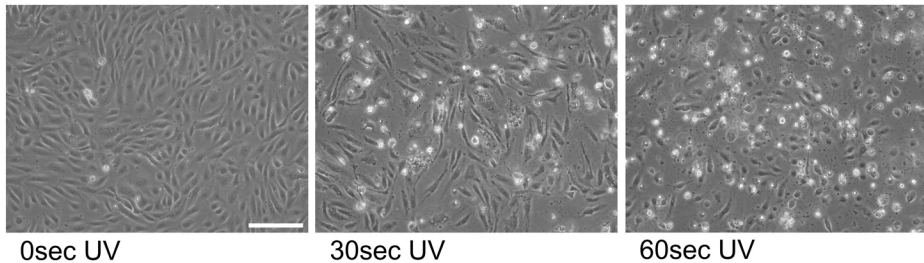

Supplement: Supplementary file 1 [file ijms-26-03155-s001.zip › Supplementary Figure S2.pdf]
